# Open Reduction and Internal Fixation of a Calcaneal Anterior Process Fracture Using a Locking Plate

**DOI:** 10.7759/cureus.18519

**Published:** 2021-10-06

**Authors:** Samuel E Cullen, Akib Khan, Chang Park, Garth Allardice

**Affiliations:** 1 Trauma and Orthopaedics, Northwick Park Hospital, London, GBR

**Keywords:** open reduction internal fixation, anterior process of calcaneum, fracture, calcaneum, missed fracture

## Abstract

Fractures involving the anterior process of the calcaneus (APC) are rare, underdiagnosed, and carry a significant increase in morbidity if not identified acutely. Identifying patients with intra-articular fracture extension is crucial as they may benefit from surgical fixation to reduce the risk of morbidity and post-traumatic osteoarthritis. There are no specific guidelines in the United Kingdom regarding the management of these fractures, and there is little evidence regarding optimal management, mainly limited to case reports and small sample observational trials. Previous reports of surgical intervention have described excision of fragments or fixation using single cancellous screws.

A 55-year-old man fell from a height of 2 metres, sustaining an APC fracture extending into the calcaneocuboid joint. This was identified on plain radiographs following a virtual fracture clinic referral from the emergency department and further investigated with computed tomography scanning.

He underwent open reduction and internal fixation with a locking T-plate and screws three weeks post-injury to restore congruence of his articular surface. Following a period of non-weight-bearing and progressive physiotherapy, he reported an excellent functional outcome six months post-operatively, measured by the American Orthopaedic Foot and Ankle Society (AOFAS) Ankle-Hindfoot Score of 90%.

In the absence of specific guidelines for these fractures, this case provides an example of good initial functional outcomes following surgical fixation using a locking plate and screws, the first such fixation of an APC fracture described in the literature. This case can also be seen as a useful reminder of the need for an index of clinical suspicion for these injuries, given that up to 40% may be missed in the emergency department. While now fairly widespread, not all hospitals will have a virtual fracture clinic system in place, meaning emergency department practitioners must be wary of these injuries before discharging patients with suspicious histories and examination findings with no follow-up. Examination techniques that may help differentiate APC fractures from ankle sprains are discussed to provide clinicians with evidence to support a suspicion of these injuries in the emergency department.

## Introduction

Fractures of the anterior process of the calcaneus (APC) are considered rare injuries. The true incidence of APC fractures is hard to judge due to the high rates of misdiagnosis and delayed diagnosis, with reports suggesting the average delay in diagnosis to be 22 weeks [1]. In fact, up to 40% of cases may be misdiagnosed as a sprained ankle initially in accident and emergency departments and around 5% of patients with a history of an ankle sprain may have a fracture of the anterior process [2]. Literature also suggests that up to 88% of APC fractures are missed on simple radiographs, which may result in underreporting of cases [3]. This is significant because delayed diagnosis or misdiagnosis have negative impacts on patient outcomes. A case series reports that fractures picked up early and treated appropriately have an average disability of 7.5 weeks, compared to an average disability of four to six months for delayed diagnoses [4].

The importance of this case is the need to identify patients that may benefit from surgical fixation to avoid future disability. Intra-articular fractures can lead to post-traumatic osteoarthritis (PTOA), which carries significant morbidity. The approach to the management of these acute injuries is to anatomically reduce and restore congruence of the articular surfaces. Patients with PTOA secondary to intra-articular calcaneocuboid joint injuries reported higher mean disability scores than those without PTOA, or with extra-articular injuries [5].

## Case presentation

A 55-year-old information technology engineer, with no past medical history, fell from a height of 2 metres from the sixth rung of a ladder onto the floor. His left foot was trapped between two rungs of the ladder during the fall, and at impact, he fell onto an inverted left foot. He had immediate pain and inability to weight bear. He presented to the emergency department where clinical assessment and radiographs were obtained.

The radiographs were initially reported as showing no fracture and the emergency physician made a diagnosis of a sprained talofibular ligament. He was placed into an ankle boot and discharged. As part of our hospital protocol, he was also referred to the trauma and orthopaedics team via a virtual fracture clinic (VFC) service.

**Figure 1:**
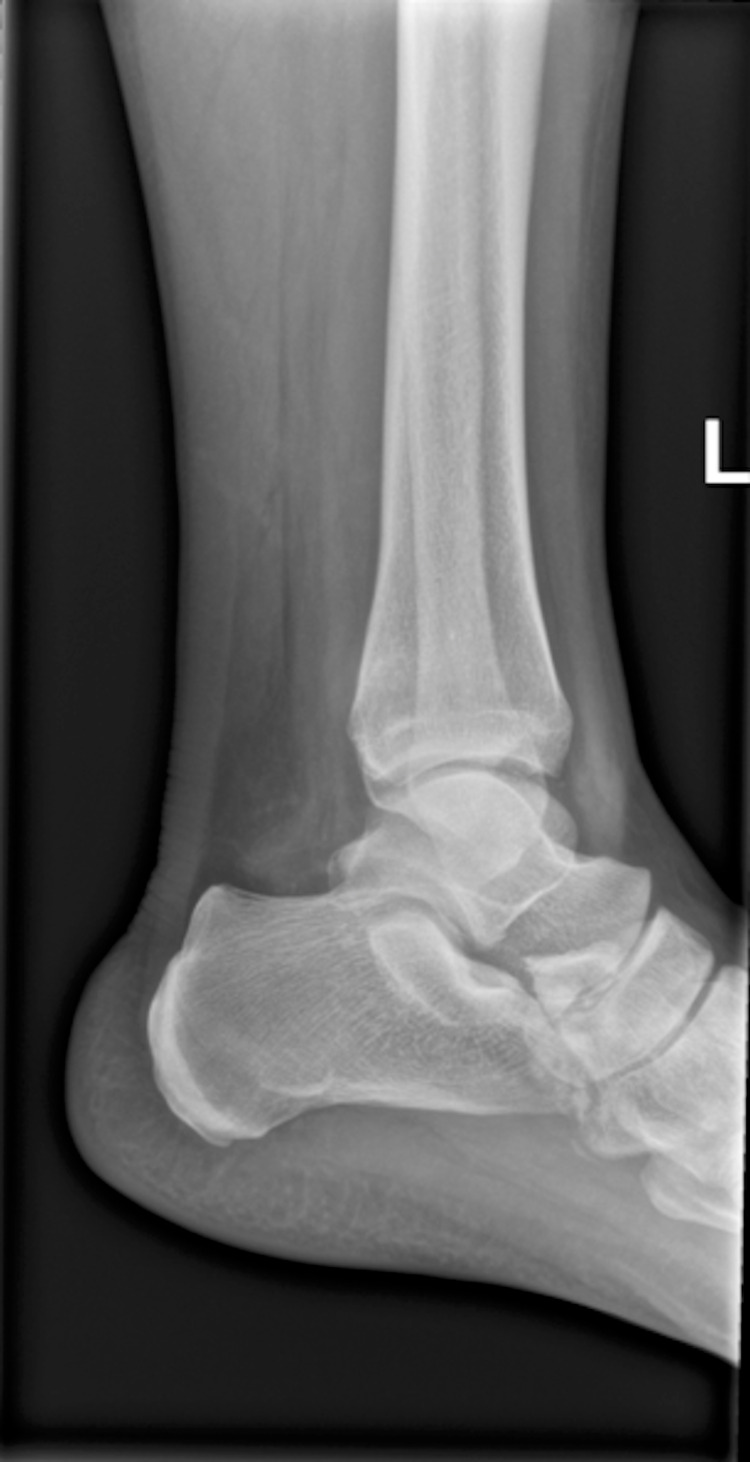
Left ankle lateral radiograph

The radiographs were reviewed in VFC the following morning and a displaced APC fracture was seen (Figure 1). An urgent clinical review was organised at the next foot and ankle specialist fracture clinic three days later.

**Figure 2:**
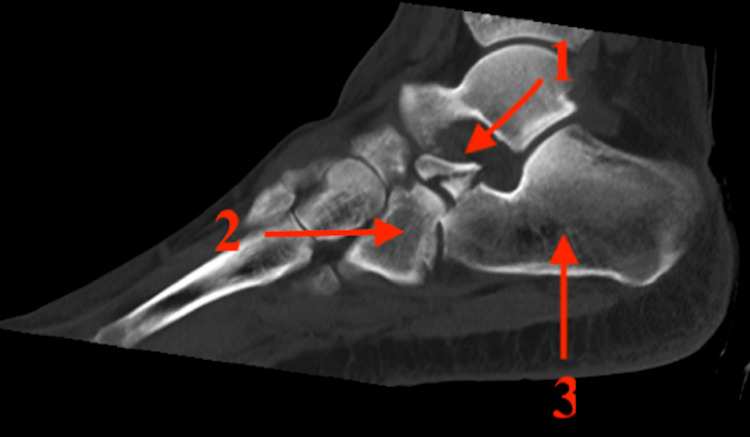
CT sagittal view of the left ankle 1: Anterior process of the calcaneus; 2: cuboid; and 3: calcaneum.

**Figure 3:**
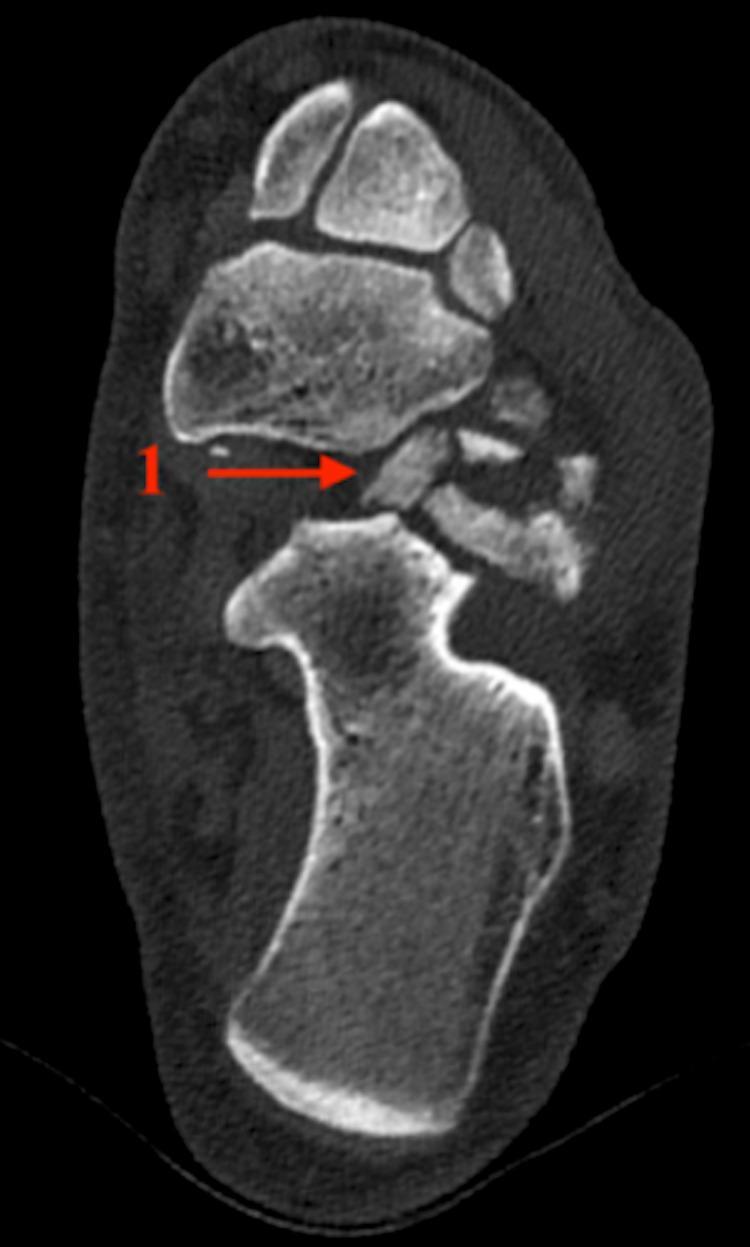
CT axial view demonstrating cuboid comminution 1: Cuboid comminution.

A CT scan of the affected calcaneus was then performed, showing a comminuted fracture of the APC involving 50% of the calcaneocuboid articular surface (Figure 2). There was also a comminuted fracture of the cuboid involving the medial surface posteriorly but there was no shortening of the bone (Figure 3).

**Figure 4:**
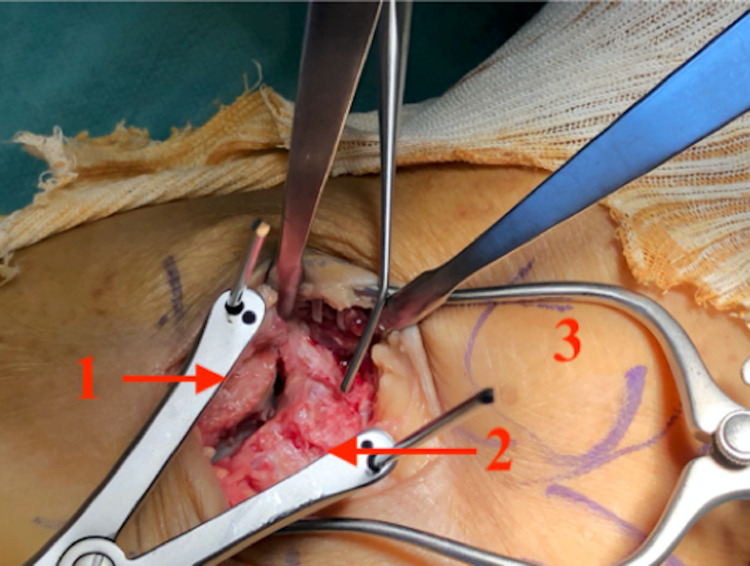
Lateral approach to ankle 1: Cuboid; 2: fracture line between the anterior process of the calcaneus (above) and calcaneus (beneath); and 3: skin markings of the fibula.

**Figure 5:**
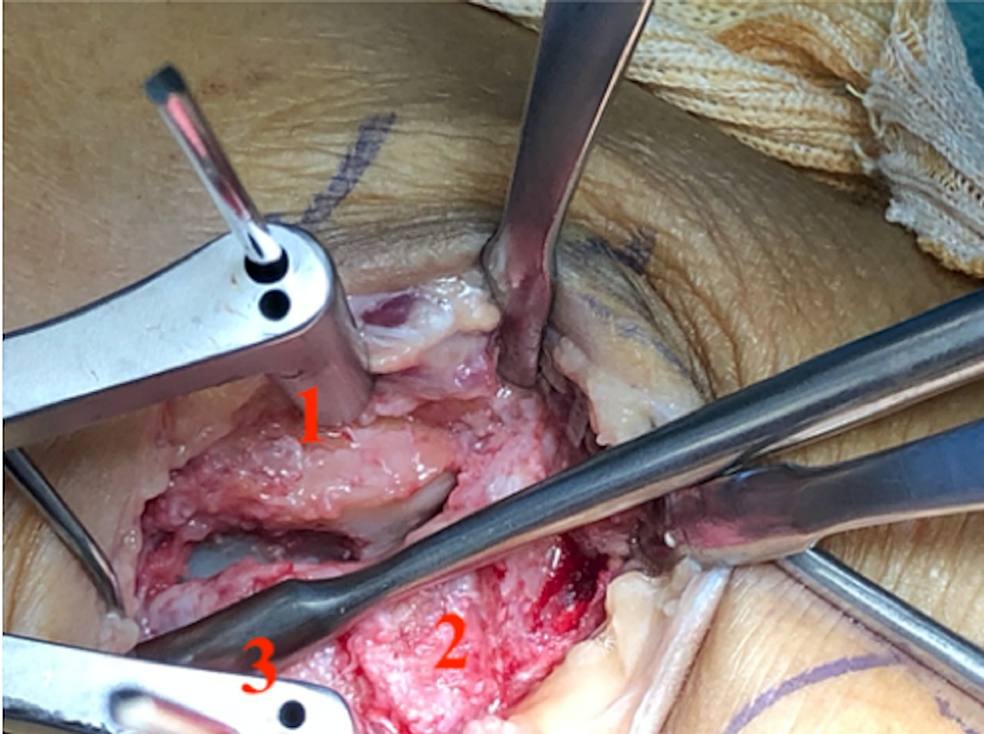
Lateral approach to ankle 1: Cuboid; 2: anterior process of the calcaneus; and 3: calcaneus.

**Figure 6:**
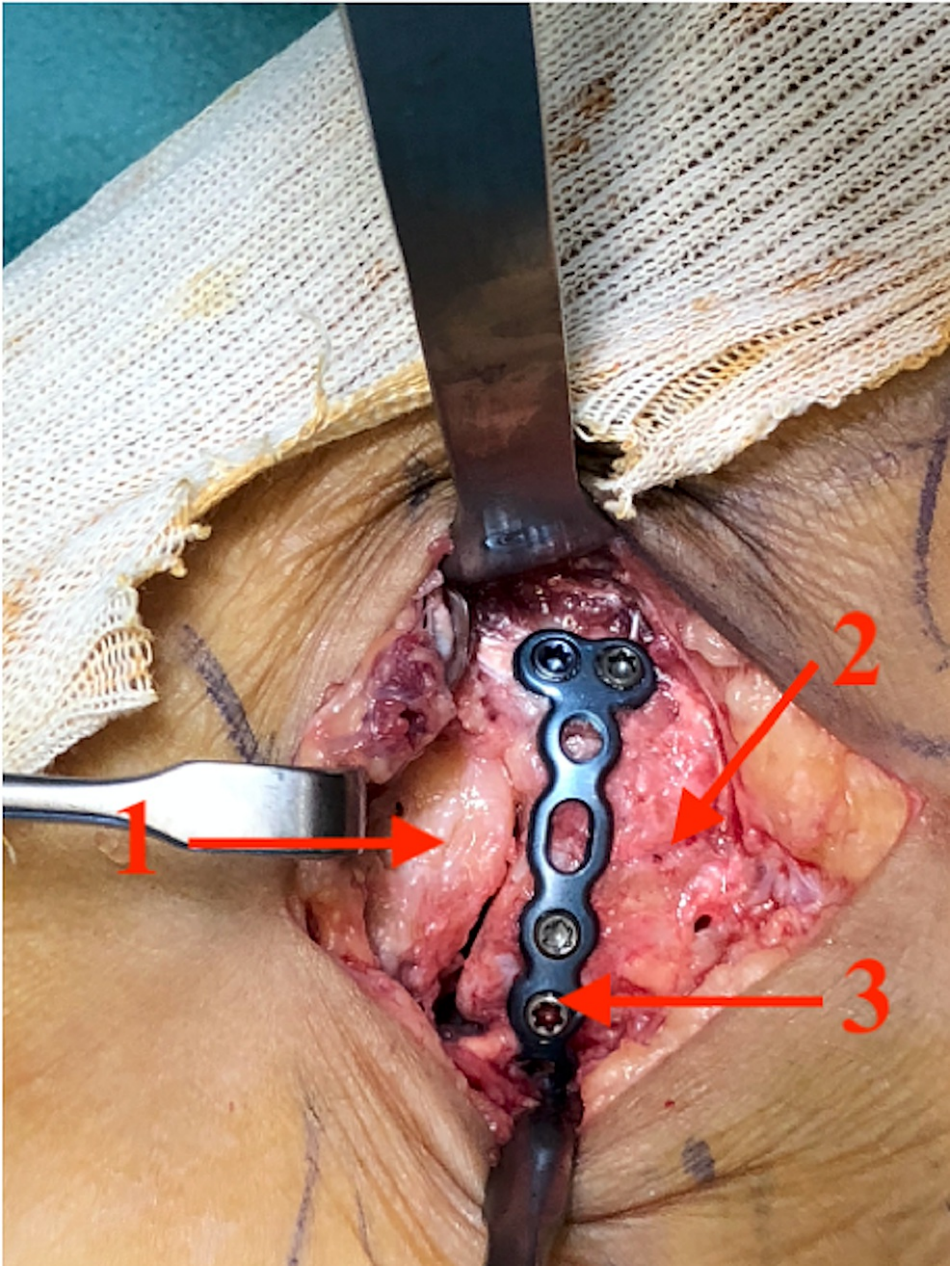
Internal fixation 1: Cuboid; 2: fracture line between the anterior process of the calcaneus and calcaneus body; and 3: moulded 1/3 tubular T-plate.

**Figure 7:**
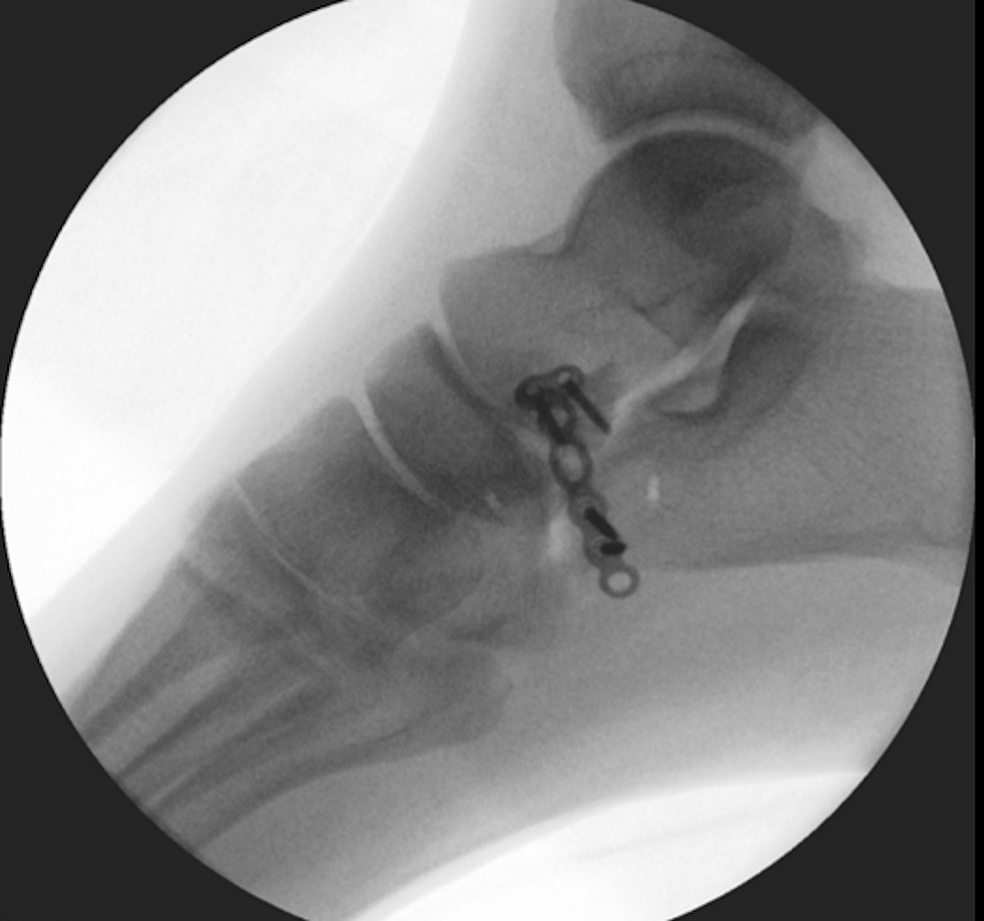
Lateral intraoperative imaging

**Figure 8:**
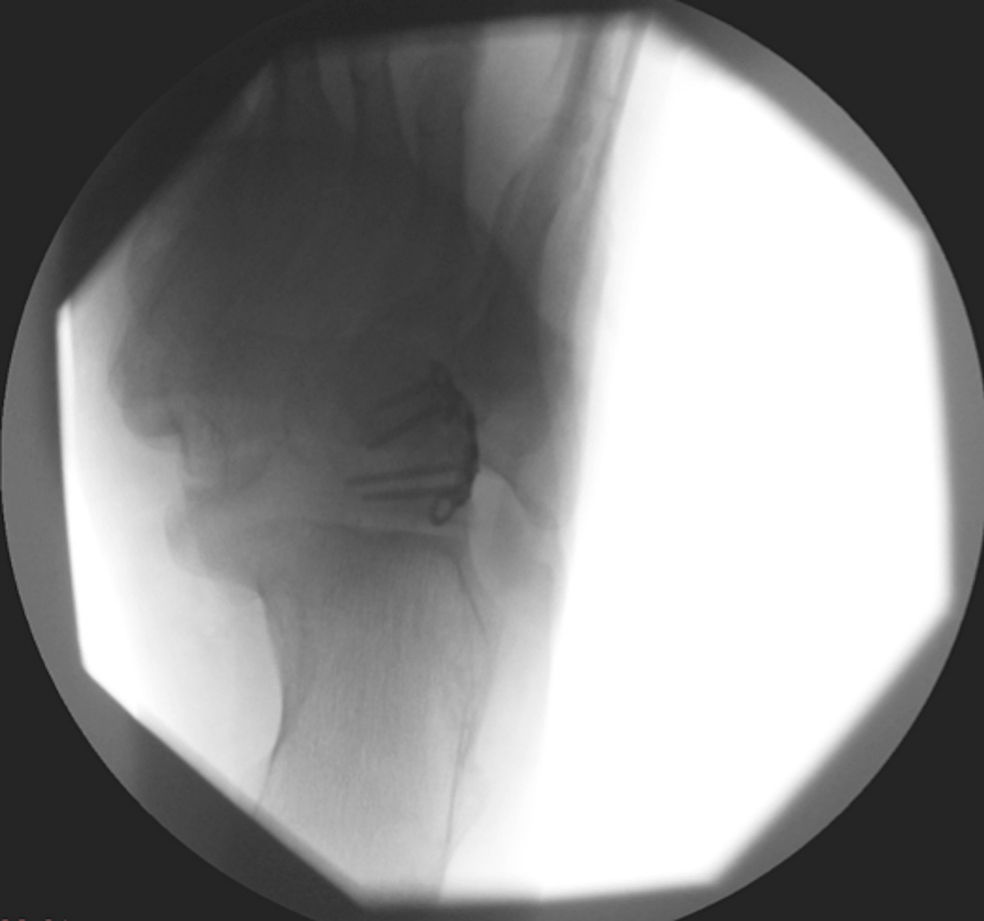
Anteroposterior intraoperative imaging

The patient was listed for an open reduction and internal fixation at three weeks post-injury once the soft tissues were less swollen. A standard lateral approach to the subtalar and calcaneocuboid joint was used. The peroneal tendons were reflected to the plantar side. Extensor digitorum brevis (EDB) and its fascia were elevated off the calcaneus to expose the joint. The fracture and all fragments were reduced anatomically (Figures 4, 5), and fixed with a moulded Arthrex (Naples, Florida, USA) 2.4 mm titanium locking T-plate (Figures 6-8).

The fixation was tested intraoperatively and the reduction was deemed stable clinically and radiologically. EDB and its fascia were reattached and then closure was done with non-absorbable sutures.

The ankle was dressed and the patient was placed in a pneumatic walker boot. He was allowed to mobilize with non-weight-bearing crutches for a period of six weeks and he could remove the boot for range of movement exercises. At the initial two-week post-operative follow-up, he was neurovascularly intact with healed surgical wounds. On review six weeks post-operatively, he had a good range of ankle and subtalar movements and was pain-free despite a small degree of stiffness.

**Figure 9:**
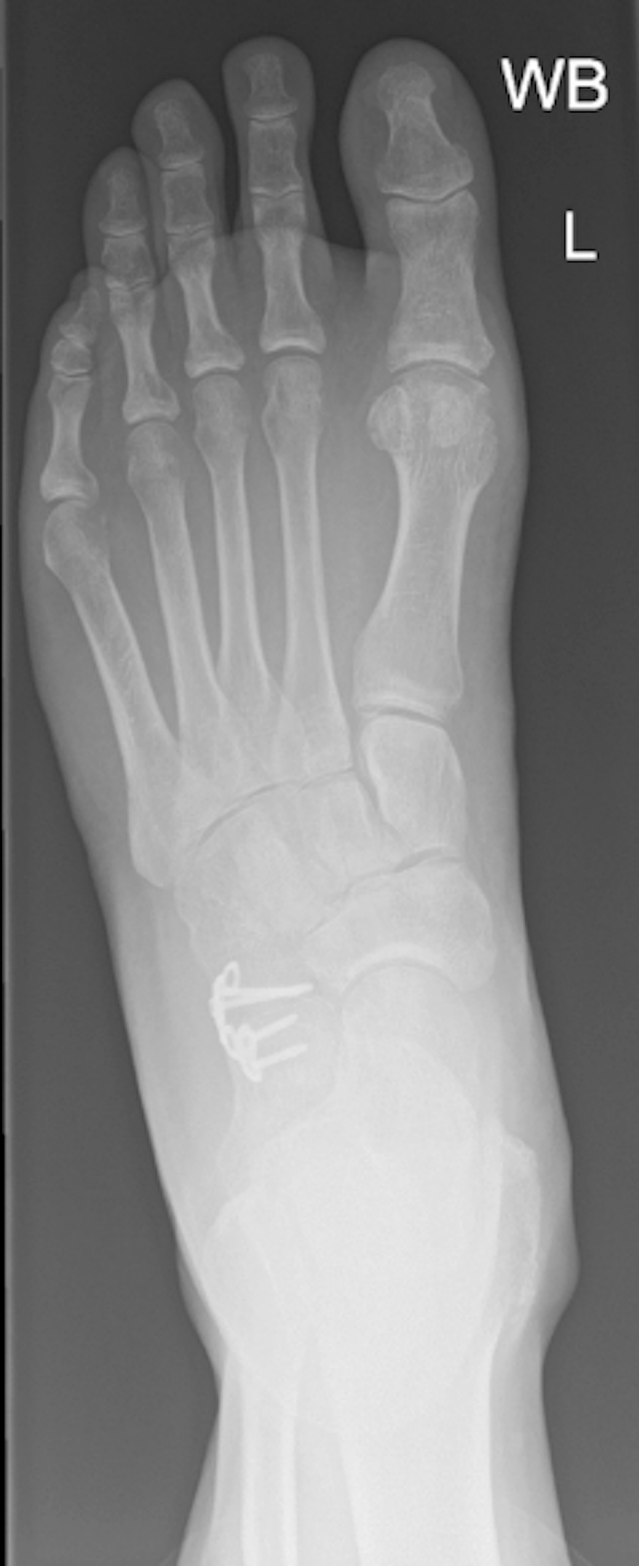
Anteroposterior radiograph of the left foot at four months post-operative

**Figure 10:**
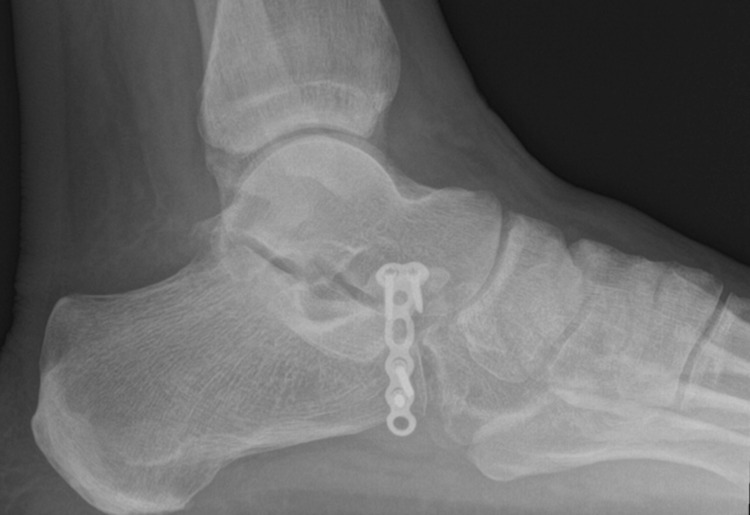
Lateral radiograph of the left foot at four months post-operative

He subsequently progressed to six weeks of partial weight-bearing and guided physiotherapy. At three months post-operative, he was able to walk fully weight-bearing and the boot was discarded. At his four-month review, the patient had continued to make excellent progress. He was pain-free, able to climb stairs, and was planning a return to the gym; he was able to swim, cycle, and walk on a cross-trainer. Repeat radiographs were obtained at this stage, which demonstrated radiological union (Figures 9, 10).

Six months post-operatively, his functional outcome and pain were assessed using the American Orthopaedic Foot and Ankle Society (AOFAS) Ankle-Hindfoot Score. While not a validated score, this is the most commonly used tool for outcome evaluation in foot and ankle surgery [6]. His score was 90 points out of 100, demonstrating excellent function and alignment while describing only very occasional hindfoot pain. An 18-month follow-up was conducted with no evidence of PTOA.

## Discussion

**Figure 11:**
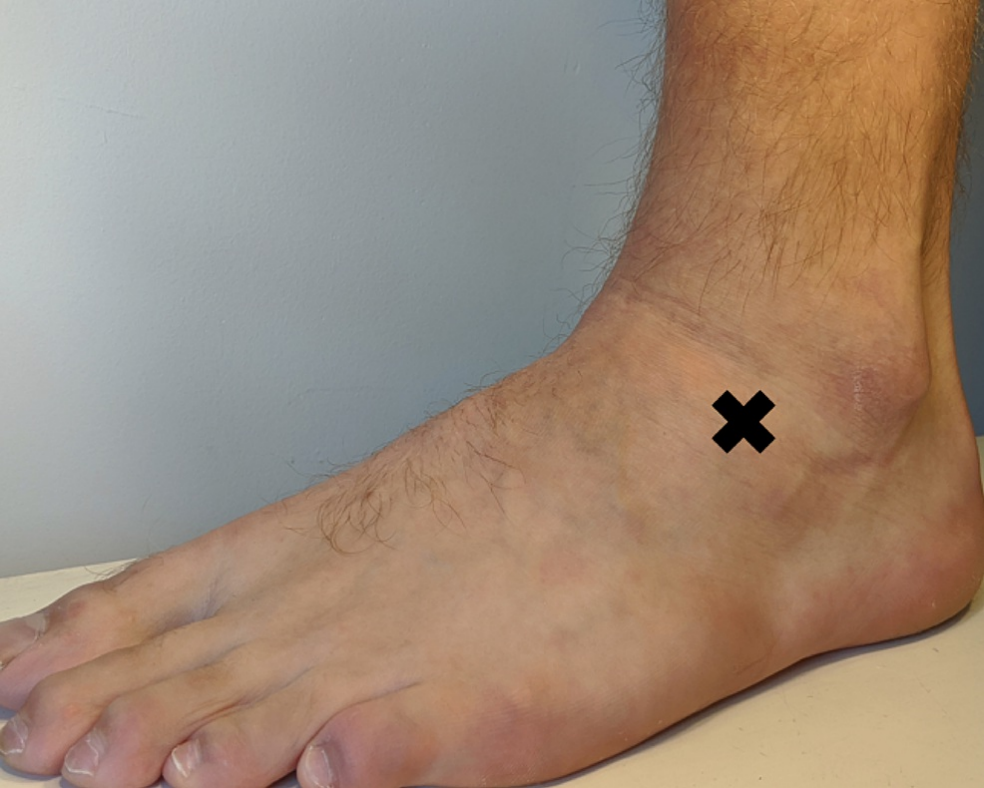
Focal tenderness 2 cm anterior to the tip of lateral malleolus is suspicious for APC fracture APC, anterior process of the calcaneus.

If an APC fracture is clinically suspected then a thorough examination is required. Clinical examination should include palpation of the dorsum of an inverted plantarflexed foot. This localizes tenderness to the sinus tarsi or calcaneocuboid joint and can often be a distinct clinical finding to generalized lateral malleolar soft tissue pain seen in ankle sprains. Another examination finding may be tenderness to palpation at a point 2 cm anterior to the tip of the lateral malleolus (Figure 11) [1,7,8].

There is little evidence regarding the optimal management of these injuries in the literature, mainly limited to case reports and small sample observational trials. Almost all fractures that do not involve the Chopart joint line are treated non-operatively with good results [1]. A case series that described some injuries extending to the calcaneocuboid joint showed they tended not to unite with immobilisation alone, leading to ongoing symptoms necessitating surgical excision of bony fragments or delayed fixation [7]. It has therefore been suggested that displaced or large acute fractures with calcaneocuboid joint involvement or displacement may be best managed with an open reduction and internal fixation [1]. A review of APC fracture management reported that a total of 17 fractures have been presented in the literature that underwent acute operative management. These operations consisted of either excision of fracture fragments [9], open reductions using single cancellous screws [10,11], or unspecified fixation. Our case report is therefore the first reported example of an isolated APC fracture fixation using a locking plate and screws.

There is a lack of both randomized comparative studies and patient-reported outcome measures (PROMs) on this topic, meaning there are currently no evidence-based guidelines to guide the management of these injuries. This case, therefore, provides a good example of how early diagnosis and plate fixation of an articular injury can give good post-operative results in an otherwise young, active, and fit patient.

## Conclusions

Important take-home messages are as follows: (1) displaced APC fractures with articular involvement may benefit from the acute open reduction and internal fixation to restore anatomy and reduce the risk of post-traumatic arthritis. (2) Use of a locking T-plate appears to be an acceptable form of open reduction and internal fixation for APC fractures, demonstrated by an excellent AOFAS Ankle-Hindfoot Score (90/100) at six months post-operatively. (3) An index of suspicion for APC fractures is important, given the morbidity associated with delayed diagnosis, especially in units with no virtual fracture clinics. This fracture was missed in the emergency department, but picked up with minimal delay due to review in our local virtual fracture clinic.
